# Rework Potential of Soy and Pea Protein Isolates in High-Moisture Extrusion

**DOI:** 10.3390/foods12132543

**Published:** 2023-06-29

**Authors:** Silvia J. E. Snel, Yasmine Amroussi, Atze Jan van der Goot, Michael Beyrer

**Affiliations:** 1 Institute of Life Technologies, University of Applied Sciences and Arts Western Switzerland, 1950 Sion, Switzerland; 2 Food Process Engineering, Agrotechnology and Food Sciences Group, Wageningen University & Research, 6708 PD Wageningen, The Netherlands

**Keywords:** meat analogues, plant protein, rheology, Lissajous plot, solubility, anisotropy

## Abstract

High-moisture extrusion (HME) is an effective process to make fibrous products that can be used as meat analogues. In this study, the effect of extrusion of already extruded products (i.e., re-extrusion) was tested with the aim to explore the potential of rework in HME. The rework of material is important because it is a route to reduce waste, which is always produced, for example during the start or at the end of a production run. Pea and soy protein isolates (PPI and SPI) were first extruded, then freeze-dried and ground, and extruded again. The visual and textural properties of the fibrous products were evaluated. Also, the rheological properties, solubility, and water-holding capacity (WHC) of the ingredients and the products after the first and second extrusion were quantified. The obtained freeze-dried powders after the first HME cycle had a reduction in solubility of 15% for PPI and 74% for SPI. Furthermore, WHC was reduced by 65% and 17% for PPI and SPI, respectively. After the second HME cycle, the reduction in solubility and WHC was augmented to 22% and 90% for PPI, and 79% and 63% for SPI. No effect on stock and loss moduli after heating and cooling were found, even after two HME cycles. SPI fibrous products did not differ in cutting strength, anisotropy index, or visual appearance after re-extrusion. Only, a decrease in hardness was detected, from 62.0 N to 51.1 N. For PPI, re-extrusion did reduce the cutting force and hardness but not the anisotropy index. It was concluded that even though HME induces a loss of solubility and WHC, this did not affect the fibrous texture formation of the protein. This means that the texture formed during HME does not depend on the process history and that rework is thus possible for fibrous products.

## 1. Introduction

High-moisture extrusion (HME) is a common method that has been used for several decades to produce fibrous products that can be used as meat analogues [1]. Soy and pea protein are often used ingredients in high-moisture extrusion to make fibrous products [2,3]. The HME process could generate waste streams during the start or end of the extrusion run. Waste produced during processing is one of the main factors that contribute to global food loss [4]. It is thus of importance to find solutions to reduce losses, for example, by reworking waste streams. HME consists of a mixing and hydration step, a thermomechanical processing step, and finally a cooling step [5]. Most proposed mechanisms behind texture formation consider the deformation of a flow in the extruder cooling die, being either laminar flow [6], phase deformation [7], or elongational flow [8]. Apart from the mechanisms considering deformation, some authors have discussed the importance of protein–protein interactions and shown that aggregates had formed after extrusion of pea and soy [9,10]. With dead-stop HME, Liu and Hsieh [11] showed that covalent binding took place in the extruder barrel before entering the cooling die. These protein–protein interactions could reduce the solubility of the protein, as has been shown for pea and soy [9,11]. However, van der Sman and van der Goot [12] argued that the high temperatures and high shear stresses during HME induce a transient protein network and that instead of measuring relative contributions of the different bonds, rheology is a more accurate method to characterize the protein melt. A closed cavity rheometer (CCR) was recently introduced to mimic extrusion-like conditions and to quantify the effects of processing on ingredient properties [13]. The CCR is able to quantify rheological properties of dense protein dispersions at high temperature and pressure through oscillatory deformation. It has been further suggested that large amplitude oscillatory shear (LAOS) in combination with Lissajous curves could reveal relevant information on the melt properties [14]. With the use of LAOS and the dissipation ratio, it was found that the elasticity of pea protein isolate (PPI) and soy protein isolate (SPI) increased after a heat treatment [15].

Noguchi [16] reported that it was possible to extrude soy protein in three to four steps by grinding the extrudate and feeding it again to the extruder, and a similar fibrous product was obtained. It was concluded that the extrudate was little affected by multiple extrusion steps, as no visual effects were observed. Furthermore, it was proposed that in HME ‘reaction’ and ‘texture formation’ should be considered independently [16]. In addition, the protein-protein reactions are expected to take place at a much smaller length scale compared to the formation of the fibrous texture [12]. This would mean that denaturation of the plant protein is less important during HME and not likely to be important for fibrous texture formation. We hypothesize that extrusion changes protein properties, for example, the solubility and water-holding capacity, and this could impact its rework potential. Therefore, this study aims to report how important these changes are for the rework ability of soy and pea by extruding them in a second step and to compare the product properties in terms of anisotropy, hardness, and cutting strength. Furthermore, the dry protein powders after the first and second steps of extrusion were compared to the isolates in terms of rheological properties and solubility and water-holding capacity.

## 2. Materials and Methods

### 2.1. Materials

Pea protein isolate (PPI, NUTRALYS^®^ F85M ) was obtained from Roquette Frères S.A. (Lestrem, France). Soy protein isolate (SPI, SUPRO 500E A^®^ 8) was obtained from Solae (St. Louis, MO, USA). PPI contained at least 83 wt% protein, and SPI 90% (N × 6.25, indicated by supplier). Powders were sieved to obtain a particle size of <400 μm (PowCN-Sif X600, CapsulCN, Ruian, China) to exclude size effects when compared with the freeze-dried powder.

### 2.2. Design of Experiments

**Figure 1:**
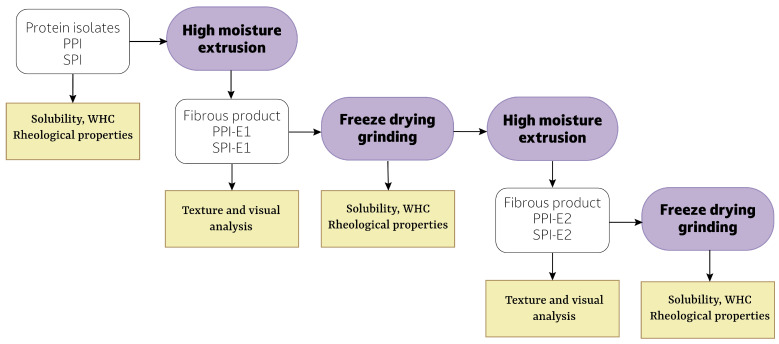
Schematic overview of the experimental design of experiments. PPI = pea protein isolate, SPI = soy protein isolate, WHC = water holding capacity.

Figure 1 shows an overview of the performed experimental steps. First, PPI and SPI were textured into fibrous products with HME (PPI-E1, SPI-E2). The obtained extrudates were freeze-dried, ground, and used again as starting material for HME. The obtained fibrous products (PPI-E2, SPI-E2) were compared to the PPI-E1 and SPI-E1 fibrous products in terms of visual appearance and texture properties. The solubility, water-holding capacity (WHC), and rheological properties of the protein isolates and freeze-dried powders were measured. All analyses are discussed in detail in the following sections.

### 2.3. High Moisture Extrusion

Protein isolates and freeze-dried extrudates (Figure 1) were extruded with an Evolum 25 twin-screw extruder (Clextral, Firminy, France). The screw diameter was 25 mm and the length/diameter ratio was 40. The extruder barrel consisted of 10 sections, which were heated to 30, 50, 70, 90, 100, 120, 130, 145, 145, and 125 °C, respectively. The rotational speed of the screws was set to 300 rpm. A rotating cooling die was attached that consists of a rotating inner cylinder and a thermo-regulated, static outer cylinder [17]. The cylinder can be further divided into two sections that can differ in their rotating speed. The rotation speed of the inner cylinder was set at 75 rpm in the first section, while the second section was kept at 0.5 rpm. The temperature of the cooling die was 85 °C. A breaker plate was placed between the extruder barrel and rotating die, which had 31 holes with a diameter of 3 mm. A twin-screw gravimetric feeder type KCM (K-Tron, Niederlenz, Switzerland) was used to feed the dry ingredients into the extruder, and water was injected in the second section with a water pump (DKM). The dry feed rate and water rate were adjusted according to the moisture content of the isolates to obtain samples with a moisture content of 58% for PPI and 62% for SPI. Throughput was 18 kg h${}^{-1}$ for all conditions.

### 2.4. Freeze-Drying of the Extrudates

Extrudates were freeze-dried and milled to facilitate feeding of those extrudates to the extruder. Freeze-drying was chosen for technical reasons and since it has limited effects on protein solubility and gelling capacity compared to other drying techniques such as oven and spray drying [18]. Extrudates were cut and collected in a sealed plastic bag. Packed extrudates were then frozen to −20 °C with a blast freezer (Electrolux, Stockholm, Sweden). Samples were then taken out of the plastic bag and transferred to a vacuum freeze-dryer (Sublimater, Zirbus, Bad Grund, Germany). The temperature of the samples was followed with temperature probes, one for each tray of samples. First, temperature was lowered to −30 °C for 3 h followed by a drying step under vacuum (0.1 mbar) for 34 h. After the samples were dehydrated, temperature and pressure were increased to 20 °C and 10 mbar. Dried samples were collected and ground with a hammer mill (Pulverizer MP, Hosokawa Alpine, Augsburg, Germany) and sieved with a vibrating sieve to obtain a particle size of <400 μm (PowCN-Sif X600, Capsulcn, Ruian, China).

### 2.5. Texture Analysis

Extrudates obtained from SPI and PPI were compared to extrudates made from freeze-dried extrudate powders of SPI and PPI. For simplicity, we will call the former SPI-E1 and PPI-E1, and the latter SPI-E2 and PPI-E1 (Figure 1). To prevent any other parameters from influencing the extrudates, all samples were prepared on the same day. Samples were defrosted overnight and compared visually by making a small inclination to aid in breaking the sample. Samples were opened in both parallel and perpendicular directions and photographed. Texture profile analysis and cutting tests were performed with a TA-XT2 texture analyzer and the Exponent Connect software (Stable Micro Systems, Surrey, UK). The texture analyzer was equipped with a 50 kg load cell and calibrated with a 1 kg weight.

#### 2.5.1. Texture Profile Analysis

The larger extrudates were cut into cylinders of 20 mm in diameter and a height of 10 mm. Samples were compressed twice with a 60 mm aluminum cylinder probe to 30% of original height with a test speed of 1 mm s${}^{-1}$ and a waiting time in between the two compressions of 5 s. Samples were measured in triplicate. The peak maximum force at first compression was taken as the hardness [19].

#### 2.5.2. Cutting Test

Cutting tests were performed in both parallel and perpendicular directions to the rotational shear flow. First, samples were cut in squares of 20 × 20 mm. Height was again 10 mm. Samples were cut up to 90% of their initial height with a Warner–Bratzler blade at a speed of 1 mm s${}^{-1}$. Anisotropy was calculated as the ratio between cutting force in parallel and perpendicular directions:

(1) $$AI_{F}=\frac{F_{\|}}{F_{⊥}}$$

### 2.6. Solubility and Water-Holding Capacity

The moisture contents of the powders were measured with a moisture analyzer (Mettler Toledo, Columbus, OH, USA). Solubility and water holding capacity (WHC) of the protein isolates and freeze-dried powders was measured in triplicate with a method reported by [20] with some minor alterations. A dispersion of 0.0125 g g${}^{-1}$ protein isolate in demineralized water was prepared and shaken overnight at 300 rpm at room temperature. Subsequently, the dispersions were centrifuged at 10,000× *g* at 21 °C for 20 min. The weight of the pellet was recorded and dried at 100 °C for at least 10 h. The solubility and WHC were calculated as follows:

(2) $$Solubility=\frac{M_{drypowder}-M_{drypellet}}{M_{drypowder}}$$

(3) $$WHC=\frac{M_{wetpellet}-M_{drypellet}}{M_{drypowder}}$$

in which $M_{drypowder}$ is the overall weight of the isolate, and $M_{wetpellet}$ and $M_{drypellet}$ are the weights of the pellet before and after drying.

### 2.7. Rheological Properties

Strain amplitude sweeps were performed with a CCR (RPA elite, TA instruments, New Castle, DE, USA). The geometry in the CCR has a radius of 2.25 mm, a maximum height of 4 mm, and a biconical opening with an angle of 3.35° to ensure homogeneous transmission of the shear stress applied. The top and bottom cones have grooves to prevent slip. The upper cone remains stationary while the lower cone oscillates. First, powders were mixed with demineralized water to obtain the same moisture content as in extrusion: 58% for PPI and 62% for SPI, calculated with the relative dry matter content of the powders. Next, approximately 5 g of the sample was placed between two plastic films in the CCR. The cavity was then sealed with a pressure of 4 bar to prevent evaporation. Samples were then heated to 30 or 145 °C for 2 min without shear. Three measuring conditions were tested: 30 °C, 145 °C, or cooled from 145 °C to 30 °C. The latter could reflect the behavior during a HME cycle, in terms of temperature profile. An amplitude strain sweep was performed from 0.1 to 1000% at 1 Hz. The yield stress and strain at the end of the linear viscoelastic (LVE) regime were defined as the point where G${}^{\prime }$ differs more than 5% from its value in the LVE regime. The flow stress and strain were defined at the crossover point, where G${}^{\prime }$ (stock modulus) is equal to G${}^{\prime \prime }$ (loss modulus). These values were used to define if the materials were mushy (low stress and low strain), brittle (low strain and high stress), rubbery (high strain and low stress), or tough (high strain and high stress) (Schreuders et al. [21]).

### 2.8. Lissajous Plots

The data obtained with the strain amplitude sweeps were further analyzed with the MITlaos software (version 2.1 beta, freeware distributed from MITlaos@mit.edu). With this software, Lissajous plots were made to visualize the response of the material to the oscillatory strain for both the elastic and viscous stress. Furthermore, the dissipation ratio was calculated [21]. The energy dissipated per unit volume in a single cycle as a function of the first-order viscous Fourier coefficient ($G_{1}^{\prime \prime }$) was calculated as:

(4) $$E_{d}=G_{1}^{\prime \prime }\gamma_{0}^{2}$$

in which $E_{d}$ is the energy dissipated and $\gamma_{0}$ the strain amplitude. For a perfect plastic material, the energy dissipated is equal to:

(5) $$E_{d}^{pp}=4\gamma_{0}\;\sigma_{max}$$

in which $\;\sigma_{max}$ is the maximum stress. The ratio between the actual energy dissipated and the energy dissipated by a perfect plastic then gives the energy dissipation ratio ϕ [22]:

(6) $$\phi =\frac{E_{d}}{E_{d}^{pp}}=\frac{\pi G_{1}^{\prime \prime }\gamma_{0}}{4\;\sigma_{max}}$$

For a perfect plastic material, this ratio would be 1. An elastic material would have a dissipation ratio of 0, and a purely viscous material would have a ratio of 0.8.

### 2.9. Standardization of Results

The relative difference between the extruded powder and extrudates was illustrated by calculating a ratio for each parameter measured. For each parameter, the obtained value was divided by the obtained value of their corresponding isolate, so SPI or PPI, or their corresponding extrudate, SPI-E1 or PPI-E1. For the corresponding sample, the value was thus taken as 1.

### 2.10. Statistical Analysis

Statistical analysis was performed with R prior to standardization of the results. It was assumed that the different factors tested in this study (protein type, processing, and temperature in the case of the rheological properties) did not interact. To confirm this, a two-way analysis of variance (ANOVA) or three-way ANOVA in the case of rheological properties was performed. First, normality and equal variance were tested with descriptive statistics. If the data were normally distributed, a two- or three-way ANOVA was performed to test if the observed differences between samples were significant (α = 0.05). Multiple comparison Tukey tests were performed to indicate which treatments were significantly different from each other (α = 0.05). If the data was not normally distributed, Welch’s ANOVA and Dunn’s tests were performed. All tests in this study were performed in triplicate.

## 3. Results

### 3.1. Extrudate Properties after Second Step HME

Extrudates were made with HME from protein isolates (PPI-E1, SPI-E1) and freeze-dried powders of the extrudates (PPI-E2, SPI-E2). The visual appearance, hardness, cutting strength, and anisotropy of cutting strength were compared.

**Figure 2:**
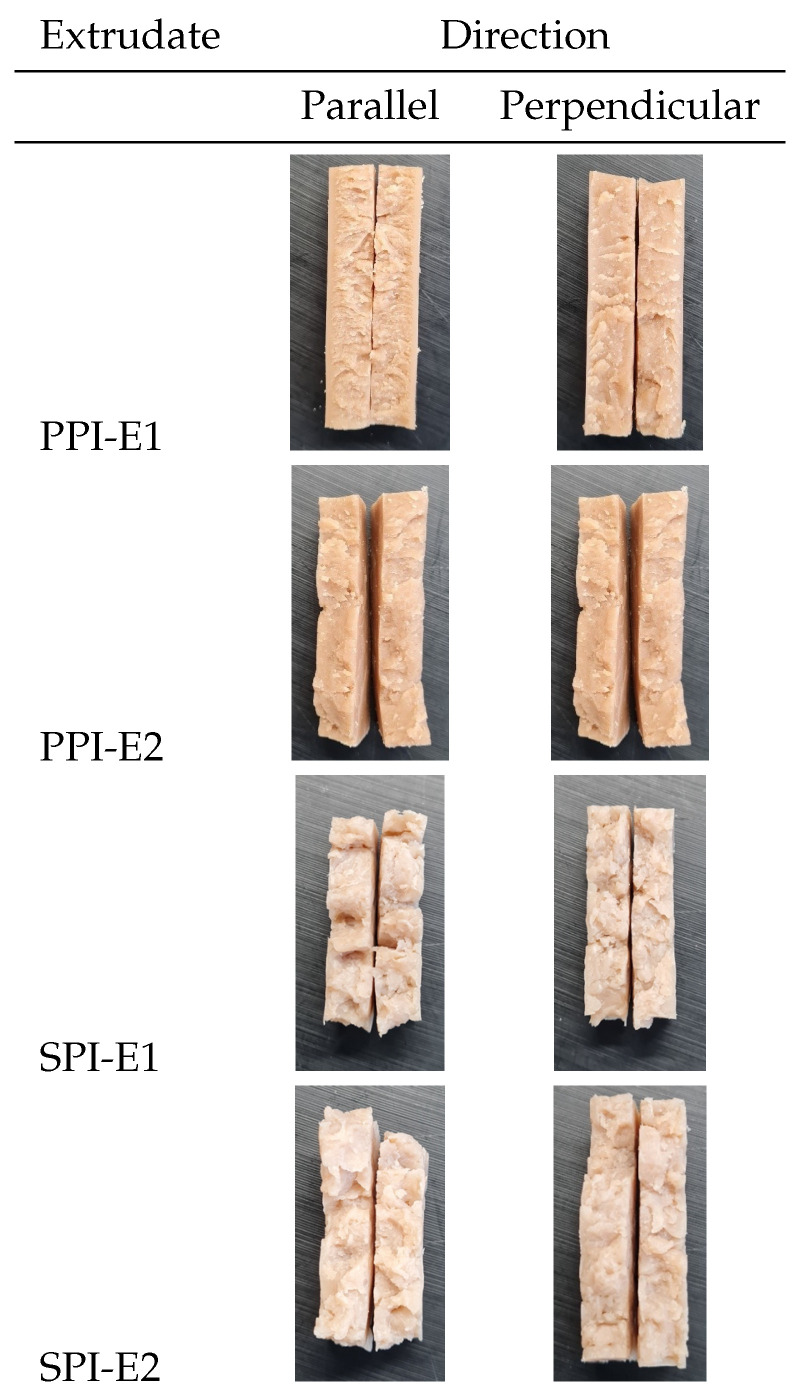
Visual appearance of extrudates from PPI (58% MC) and SPI (62%). A small inclination was made to allow breaking of the extrudates in parallel and perpendicular direction to the shear flow in the rotating cooling die. Samples -E1 were made from the isolates (PPI, SPI), and samples -E2 were made from the freeze-dried and ground -E1 extrudates.

The samples prepared from protein isolates and freeze-dried powders of the extrudates did not differ in their visual appearance for both SPI and PPI (Figure 2). In general, it was noted that SPI samples looked more inhomogeneous when broken in the parallel direction, while for PPI no clear difference was observed between the parallel and perpendicular direction of breaking. This observation was made for products obtained after the first and second extrusion steps.

**Figure 3:**
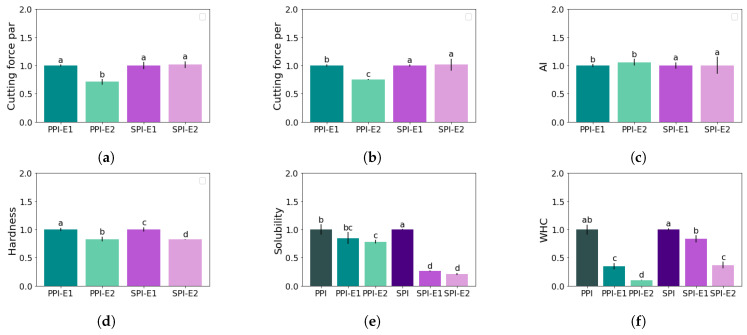
Relative change of extrudate parameters obtained from a cutting test (**a**–**c**), texture profile analysis (**d**), and relative solubility (**e**) and WHC (**f**) of the powders after HME after a first (-E1) and second (-E2) HME step as compared to PPI and SPI (Table 2). Values are reported as averages with standard deviations (black bars); letters indicate significant groups; *n* = 3. A relation was found between HME cycle and protein type for cutting force in both par and per direction, solubility, and WHC (α < 0.05).

The hardness, cutting force in both parallel and perpendicular directions, and the calculated anisotropy index were set to 1 for the PPI-E1 and SPI-E1 extrudates (Table 1). Then, the obtained values of the PPI-E2, and SPI-E2 samples were divided by the values obtained for PPI-E1, and SPI-E1, to show their relative difference (Figure 3). For PPI, cutting force decreased in both parallel and perpendicular directions after the second HME step, and a similar anisotropy index was thus found (Figure 3a–c). The cutting force in both directions was not altered for SPI after a second HME step, resulting in a similar anisotropy index. It can further be observed that the hardness of the PPI-E2 and SPI-E2 samples was slightly lower compared to PPI-E1 and SPI-E1 (Figure 3d).

### 3.2. Effect of HME on Protein Properties

**Figure 4:**
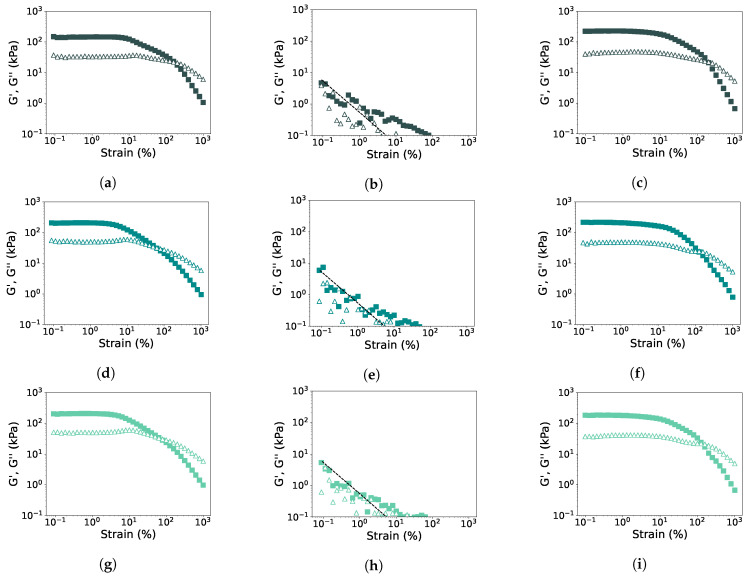
Strain sweeps (f = 1 Hz) of PPI (**a**–**c**), PPI-E1 (**d**–**f**), and PPI-E2 (**g**–**i**) at 30 °C (**a**,**d**,**g**), at 145 °C (**b**,**e**,**h**), and heated to 145 °C and cooled to 30 °C (**c**,**f**,**i**). G${}^{\prime }$: closed symbols and G${}^{\prime \prime }$: open symbols. The black dotted line in the measurement at 145 °C represents the torque limits of the CCR.

**Figure 5:**
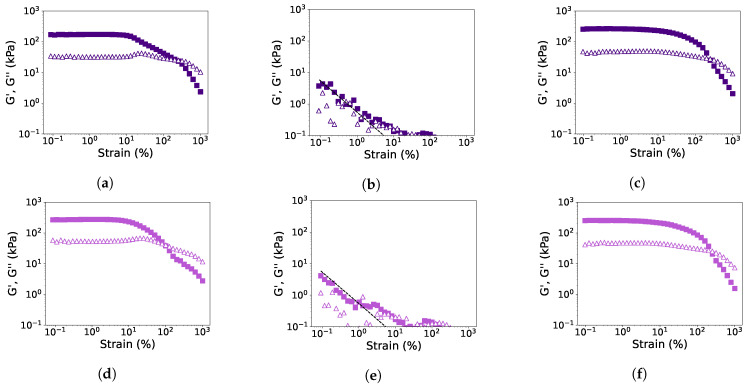

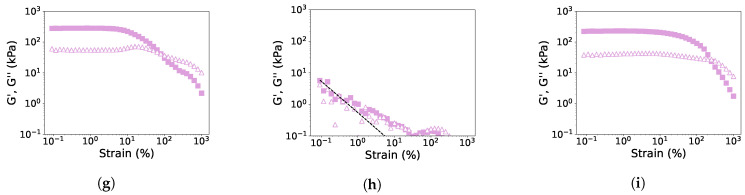
Strain sweeps (f = 1 Hz) of SPI (**a**–**c**), SPI-E1 (**d**–**f**), and SPI-E2 (**g**–**i**) at 30 °C (**a**,**d**,**g**), at 145 °C (**b**,**e**,**h**), and heated to 145 °C and cooled to 30 °C (**c**,**f**,**i**). G${}^{\prime }$: closed symbols and G${}^{\prime \prime }$: open symbols. The black dotted line in the measurement at 145 °C represents the torque limits of the CCR.

**Figure 6:**
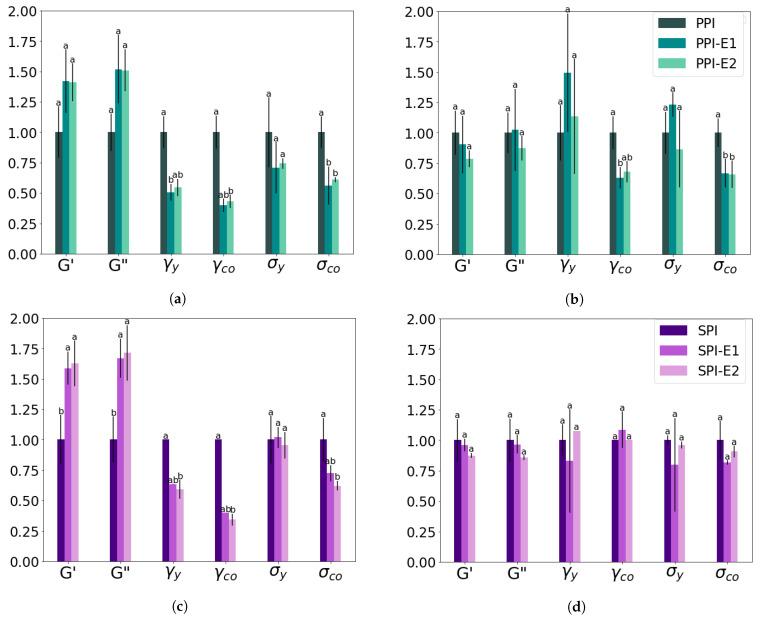
Relative change of rheological parameters obtained from a strain sweep after a first (-E1) and second (-E2) HME step as compared to PPI (**a**,**b**) and SPI (**c**,**d**) (Table 3). The strain sweeps were performed at 30 °C (**a**,**c**) and after heating to 145 °C and cooling to 30 °C (**c**,**d**). Values are reported as averages with standard deviations (black bars); letters indicate significant groups per variable; *n* = 3.

**Figure 7:**
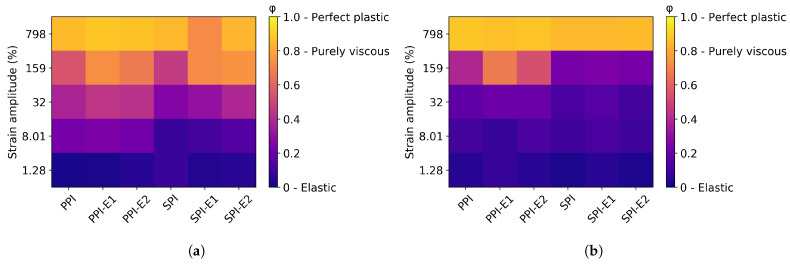
Heat map of the dissipation ratio ϕ at different strain amplitudes for the powders before (SPI, PPI) and after one (-E1) and two (-E2) HME cycles, measured at 30 °C, (**a**) and after heating to 145 °C followed by cooling to 30 °C (**b**).

Apart from the comparison between extrudates after a second HME step, the obtained freeze-dried powders were also analyzed. Extrudates obtained after the first (called -E1) and second (-E2) HME were freeze-dried, ground, and sieved, and their properties were compared to the protein isolates (PPI, SPI) in terms of solubility, WHC (Table 2, Figure 3e,f), and rheological properties (Table 3, Figure 4, Figure 5, Figure 6 and Figure 7).

Solubility and WHC of PPI were slightly lower than the solubility and WHC of SPI (Table 2). The obtained powders from freeze-dried extrudates, PPI-E1 and SPI-E1, had a lower solubility compared to PPI and SPI (Figure 3e). For PPI, this reduction was not significant. The powders obtained from the second-step extrudates, PPI-E2 and SPI-E2 had again a lower solubility, but this additional decrease was not significantly different from the PPI-E1 and SPI-E1 samples.

The WHC of the PPI-E1 and SPI-E1 samples was significantly lower compared to PPI and SPI (Figure 3f). The second HME step significantly reduced the WHC further.

Overall, the effect of HME on solubility and WHC was similar for both PPI and SPI, although the solubility was more drastically reduced for SPI and the WHC for PPI. It seems that HME leads to additional cross-links which impact the powder properties. With the use of cross-linking and reducing agents, it was found that more cross-links lead to lower WHC for whey protein [23]. The WHC could be used to predict the maximum moisture content during HME. For example, PPI-E1 had a WHC of 3.3 ± 0.4 g g${}^{-1}$, and extrudates had a dry matter content of 42%. The 42 g PPI-E1 could thus hold 140 g water, which translates to a maximum moisture content of 77%, which is still higher than the 58% in the extrudate. However, PPI-E2 had a WHC of 1.0 ± 0.1 g g${}^{-1}$, which would result in a maximum moisture content of 50%. Therefore, extruding PPI-E2 in a third HME cycle might become problematic.

Rheological properties were examined with a strain amplitude sweep at 30 °C, 145 °C, and heating to 145 °C followed by cooling to 30 °C (Figure 4 and Figure 5). The measured G${}^{\prime }$ and G${}^{\prime \prime }$ at 145 °C were found to be close to or even below the torque limits of the CCR, and thus it was not possible to determine the LVE-regime, yielding, and cross-over point for the measurements at this temperature. From the other strain amplitude sweeps, G${}^{\prime }$ and G${}^{\prime \prime }$ in the LVE-regime, yield strain and stress, and flow strain and stress were determined (Table 3). The strain and stress at the yield and flow point give an indication of the texture of the material, being either mushy, rubbery, tough, or brittle [21]. The alterations of these parameters after the first and second HME steps were again calculated relative to PPI and SPI (Figure 6). It can be seen that the HME step increased the G${}^{\prime }$ and G${}^{\prime \prime }$ measured at 30 °C for both PPI and SPI, and this remained constant after the second step. After heating to 145 °C and cooling to 30 °C, no significant changes were found for both PPI and SPI, although G${}^{\prime }$ and G${}^{\prime \prime }$ decreased slightly with each step.

The lower yield stress and strain of PPI samples compared to SPI, indicated a more mushy material for PPI compared to the brittle SPI (Table 3). Both the yield strain and flow strain decreased significantly for both PPI and SPI measured at 30 °C (Figure 6a,c). In other words, after an HME step, the isolates will yield and flow at a lower strain, and this might affect the flow behavior in the extruder barrel. After heating to 145 °C and cooling to 30 °C, PPI samples were less affected, and yield strain and stress even increased after the first HME step (Figure 6b). SPI became slightly more brittle after the first HME cycle, seen by the lower yield strain, but after the second cycle, a similar stress and strain value was found as for the non-extruded SPI (Figure 6d). So, breakdown of the network structure seems to take place, but it can (partly) recover.

The flow stress and strain were determined at the cross-over point of the amplitude sweeps. Again, a lower flow strain and stress were observed for PPI compared to SPI, indicating that SPI is a more tough material (Table 3). The first HME step reduced the flow strain and stress for both PPI and SPI, indicating a more mushy or brittle material (Figure 6a,c). After the second HME step, the strain and stress were not reduced further. After heating to 145 °C and cooling to 30 °C, both the flow strain and stress were less affected, but still, a reduction was observed for PPI samples (Figure 6b,d). Interestingly, the cross-over stress and strain of SPI were not changed after two cycles of extrusion after heating and cooling, indicating that the SPI samples remained tough.

Lissajous curves were made from the strain amplitude sweeps (Appendix A), and dissipation ratios were calculated (Figure 7). At 30 °C, all samples were in the elastic regime at low strain amplitude and became more viscous with increasing strain amplitude. For both PPI and SPI, the point where the samples became viscous was at a lower strain amplitude after the first HME cycle. A second HME cycle did not seem to affect this further. When the samples were first heated to 145 °C and then cooled again to 30 °C, both PPI and SPI remained elastic at higher strain amplitudes compared to the unheated samples. This increase in elasticity has been found before for both pea and soy [21]. The first and second HME cycles made the PPI samples slightly more viscous at higher shear, but almost no difference was observed for the SPI samples.

## 4. Discussion

In this study, the rework potential of fibrous products was tested by repetitive extrusion of protein isolates. This was accomplished by comparing the relative change of the obtained fibrous products in terms of hardness, cutting strength, and anisotropy. For improved comparability, extrudates were freeze-dried and ground before the second HME step. The properties of the obtained powder after the first and second HME steps were compared to the untreated protein isolate in terms of solubility, WHC, and rheological properties.

The effect of repetitive extrusion on the extrudate properties was small, especially for SPI. Visually, no differences were found between samples after the first and second HME steps. For soy protein, this was observed before, as no visual difference was reported for defatted soy protein after three HME steps [24]. PPI extrudates had a lower anisotropy index compared to SPI, which has been observed previously [17]. For SPI samples the cutting force and anisotropy indexes were not affected by the second HME step. Only the hardness was slightly decreased. For PPI, the cutting force and hardness were decreased, however, the anisotropy index remained the same. This then confirms that soy can be extruded in several cycles without altering product properties, as was suggested before [24]. The ability to extrude SPI and PPI in several HME cycles is remarkable, as we see that ingredient properties (solubility, WHC) changed. This could be explained because some changes are less relevant; for example, the WHC was still sufficient, and other changes were partly reversible through heating. The changes in rheological properties became smaller after heating, suggesting the breaking and reforming of bonds upon heating. The hydrophobic bonds in proteins are broken at temperatures of 130–140 °C [7]. Furthermore, the high shear rates in the extruder barrel could lower the activation energy for breaking the disulfide bonds [25]. Each HME cycle reduced the solubility and the WHC of both PPI and SPI, even if the reduction in solubility was only significant after two HME cycles for PPI. The decrease in solubility after HME has been reported previously for both PPI [9] and SPI [11], although both studies used reducing buffers to break specific bonds between the proteins. Isobe and Noguchi [24] also found a reduction of soy protein solubility after each HME step. The reduced solubility and WHC were probably caused by the formation of insoluble aggregates during HME. Fang et al. [10] performed sodium dodecyl sulfate-polyacrylamide gel electrophoresis (SDS-PAGE) before and after extruding SPI and observed a reduction in the intensity of specific bands, which they attributed to aggregate formation. Similarly, Osen et al. [9] saw a reduction of specific bands in SDS-PAGE after HME of PPI and came to a similar conclusion. The decrease in WHC could also reflect a higher density of cross-links [23].

The formation of aggregates could also explain observed changes in rheological properties when measured at 30 °C. Remarkably, these changes became smaller after heat treatment was performed. Possibly, the heating and cooling step allowed the proteins to rearrange in a more optimal way. For pea protein, it has been found previously that a low cooling rate allowed pea vicilin to make more optimal interactions (O’Kane et al. [26]). O’Kane et al. [26] showed that the elastic modulus of soy protein gels followed the same trajectory during reheating and subsequent cooling between 85 and 25 °C, even at a higher cooling rate. This then further confirms the found reversibility of soy during extrusion [16]. In this study, rework was tested by freeze-drying the extrudates and feeding them as a powder to the extruder. We are confident that rework is also possible using other mild drying techniques. In the case rework is combined with native ingredients, it might be possible to add the rework without a drying step. The latter has been proven for extrudates made from soy flour [24].

## 5. Conclusions

SPI and PPI were extruded twice to test rework potential. First, extrudates for both protein isolates were obtained with HME and subsequently, these extrudates were freeze-dried and ground for a next HME cycle. HME led to a reduction of solubility and WHC of proteins, probably caused by protein–protein interactions and aggregate formation. A second HME cycle reduced solubility and WHC further. However, after heating, the rheological properties were not significantly different after HME. This was explained by the weakening of protein–protein interactions upon temperatures of 145 °C. In HME, the weakening of the bonds will be further achieved by the high shear stresses. We, therefore, hypothesize that protein aggregates can be formed and aligned in a repetitive, reversible manner, and fibrous textures can be formed again. This hypothesis was confirmed by the similarity in structural and textural properties of the fibrous products of the first and second HME cycle. For both PPI and SPI, the visual appearance of the extrudates was not altered. Even though the hardness and cutting strength of PPI were slightly reduced, visual aspects and anisotropy of PPI fibrous products remained similar after the second HME step. It is concluded that the reactions taking place in HME can be viewed as separate from the texture formation. SPI could be seen as a thermoplastic reversible material, and even PPI was not affected largely by HME. This thus concludes that rework is possible for both PPI and SPI.

## Figures and Tables

**Table 1:** Hardness (N), cutting force (N) in both parallel (par) and perpendicular (per) direction, and the anisotropy index calculated as the ratio between the two cutting forces for PPI-E1 (58% MC) and SPI-E1 (62% MC) extrudates, letters indicate significant groups, *n* = 3.

Extrudate	Hardness (N)	Cutting Force Par (N)	Cutting Force per (N)	AI (-)
PPI-E1	116 ± 3.5 ${}^{a}$	14.3 ± 0.2 ${}^{a}$	15.1 ± 0.3 ${}^{b}$	1.1± 0.03 ${}^{b}$
SPI-E1	62.0 ± 2.5 ${}^{b}$	13.6 ± 0.8 ${}^{a}$	22.1 ± 0.4 ${}^{a}$	1.6± 0.1 ${}^{a}$

**Table 2:** Solubility, and WHC for PPI and SPI before and after extrusion. After extrusion, extrudates were freeze-dried, ground, and sieved (-E), this powder was extruded, freeze-dried, ground, and sieved again (-E2). Values are averages ± standard deviation, and letters indicate significant groups, *n* = 3.

Powder	Solubility (g g${}^{-1}$)	WHC (g g${}^{-1}$)
PPI	0.30 ± 0.03 ${}^{b}$	9.6 ± 0.8 ${}^{ab}$
SPI	0.47 ± 0.00 ${}^{a}$	11 ± 0.2 ${}^{a}$

**Table 3:** Storage modulus (G${}^{\prime }$), loss modulus (G${}^{\prime \prime }$), yield strain ($\gamma_{y}$), yield stress ($\sigma_{y}$), flow strain ($\gamma_{co}$), and flow stress ($\sigma_{co}$) for PPI and SPI before and after extrusion. After extrusion, extrudates were freeze-dried, ground, and sieved (-E1), this powder was extruded, freeze-dried, ground, and sieved again (-E2). G${}^{\prime }$, $\gamma_{y}$, and $\gamma_{co}$ were determined from strain amplitude sweeps. Values are averages ± standard deviation, and letters indicate significant groups, *n* = 3.

Powder	Temperature (°C)	G${}^{\prime }$ ($10^{2}$ kPa)	G${}^{\prime \prime }$ ($10^{2}$ kPa)	$\gamma_{y}$ (%)	$\sigma_{y}$ (kPa)	$\gamma_{\mathrm{co}}$ (%)	$\sigma_{\mathrm{co}}$ (kPa)
PPI	30	1.4 ± 0.3 ${}^{b}$	0.3 ± 0.0 ${}^{a}$	4.8 ± 0.5 ${}^{bc}$	6.9 ± 0.5 ${}^{b}$	137 ± 19 ${}^{b}$	48 ± 5.1 ${}^{b}$
	145-30	2.2 ± 0.4 ${}^{ab}$	0.5 ± 0.1 ${}^{a}$	3.3 ± 0.6 ${}^{c}$	7.1 ± 1.0 ${}^{b}$	173 ± 24 ${}^{ab}$	62 ± 5.9 ${}^{b}$
SPI	30	1.7 ± 0.3 ${}^{ab}$	0.3 ± 0.1${}^{a}$	8.0 ± 0.0 ${}^{a}$	14 ± 2.1 ${}^{ab}$	200 ± 0.0 ${}^{a}$	75 ± 11 ${}^{ab}$
	145-30	2.5 ± 0.4 ${}^{a}$	0.5 ± 0.1 ${}^{a}$	6.5 ± 1.2 ${}^{b}$	16 ± 1.1 ${}^{a}$	200 ± 0.0 ${}^{a}$	105 ± 13 ${}^{a}$

**Figure A1:**
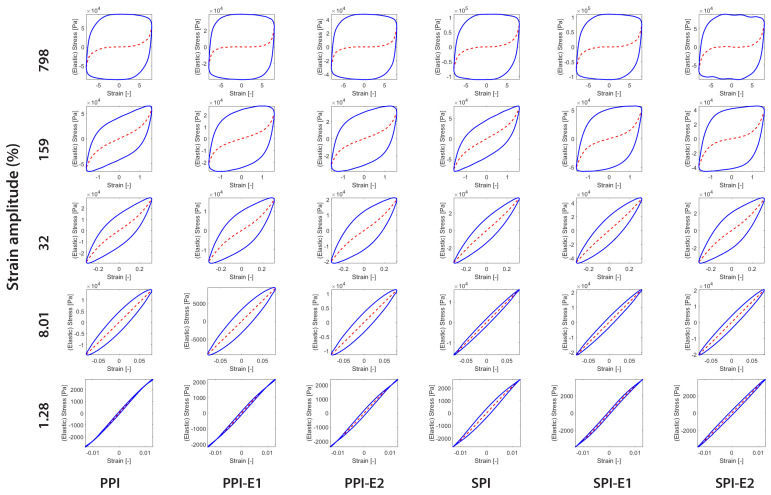
Elastic lissajous curves of the stress versus strain amplitude at 30 °C. Solid lines represent normalized stress and dashed lines the elastic stress.

**Figure A2:**
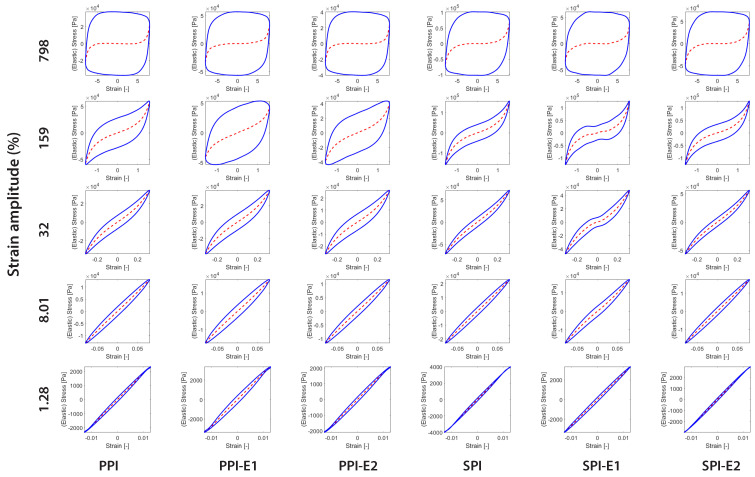
Elastic lissajous curves of the stress versus strain amplitude of samples heated to 145 °C and cooled to 30 °C. Solid lines represent normalized stress and dashed lines represent the elastic stress.

**Figure A3:**
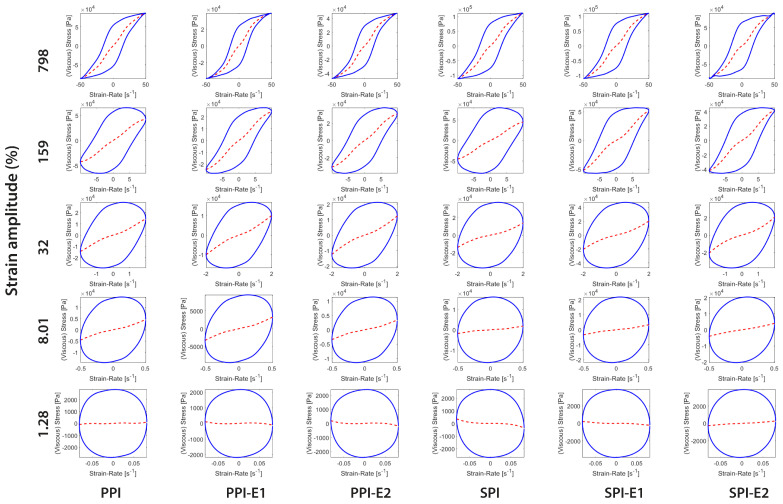
Viscous lissajous curves of the stress versus strain amplitude at 30 °C. Solid lines represent normalized stress and dashed lines represent the elastic stress.

**Figure A4:**
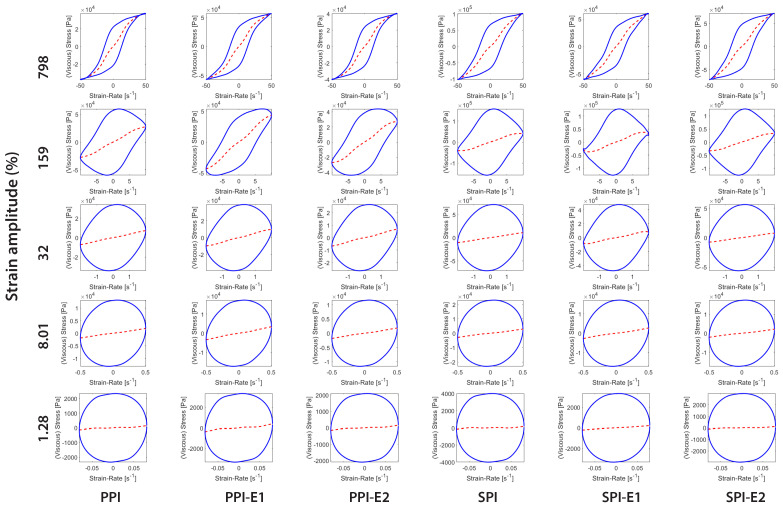
Viscous lissajous curves of the stress versus strain amplitude of samples heated to 145 °C and cooled to 30 °C. Solid lines represent normalized stress and dashed lines the elastic stress.

## Data Availability

Data available on request from the corresponding author.
